# Adaptive Algal Cultivation Enabled by a Monthly Biomass Forecasting System

**DOI:** 10.1002/bit.70120

**Published:** 2025-12-05

**Authors:** Hongxiang Yan, Song Gao, Mark S. Wigmosta, Andre M. Coleman, Ning Sun, Michael H. Huesemann

**Affiliations:** ^1^ Energy and Environment Directorate Pacific Northwest National Laboratory Richland Washington USA; ^2^ Marine Sciences Laboratory Pacific Northwest National Laboratory Sequim Washington USA; ^3^ Department of Civil and Environmental Engineering University of Washington Seattle Washington USA

**Keywords:** Huesemann Algae Biomass Growth Model, monthly biomass forecasting, *Picochlorum celeri*, pond water depth, strain selection, *Tetraselmis striata*

## Abstract

Microalgae offer a promising pathway for sustainable biofuel and bioproduct development, but outdoor cultivation is highly sensitive to environmental variability. To address this, the authors present an experimental monthly biomass forecasting system designed to guide operational decisions such as strain selection and pond water depth. Using the Biomass Assessment Tool (BAT) coupled with two forecasting approaches, a climatology‐based method using data from Phase 2 of the North American Land Data Assimilation System (NLDAS‐2) and three models from the North American Multi‐Model Ensemble (NMME), the authors evaluated biomass production strategies for two high‐performing algal strains (*Picochlorum celeri* and *Tetraselmis striata*) across four pond depths (15–30 cm) from 2020 to 2024 in Arizona. One of the NMME models achieved the highest selection accuracy, correctly identifying the optimal strain and pond depth in 84% of the months, with model accuracies across the NMME suite ranging from 74% to 84%. In comparison, the NLDAS‐2 climatology‐based approach achieved a 78% accuracy. Strain selection was consistently more accurate than pond depth selection across all methods, with one NMME model and the NMME multi‐model ensemble achieving up to 92% accuracy in strain prediction. Simulation results show that forecast‐informed approaches increased average biomass yields by 15% over the current State‐of‐Technology strategy, with gains exceeding 40% in certain months. These results highlight the potential of forecast‐guided strategies to enhance biomass production and enable more adaptive, weather‐resilient microalgae cultivation. The system is scalable to additional strains and geographic regions, offering a flexible tool for advancing sustainable algal production under increasingly variable environmental conditions.

## Graphical abstract

The developed monthly algal forecasting system used to predict biomass production for two algal strains (A and B) at two pond water depths (low and high). The highest predicted production, achieved with strain A at a low pond water depth, is selected as the cultivation strategy to guide the next month's algal growth.

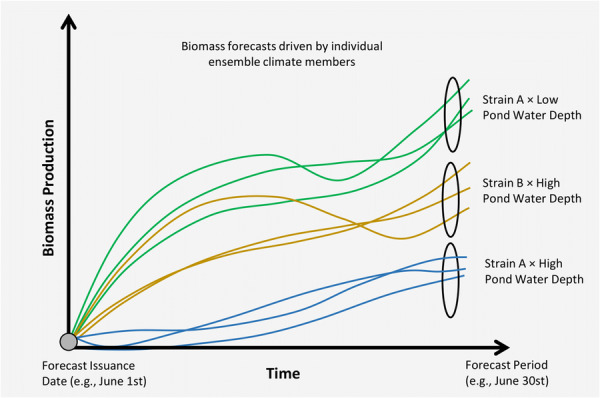

## 1 Introduction

Microalgae have garnered significant attention as a sustainable resource for producing biofuels, bioproducts, and proteins, owing to their rapid growth rates, high photosynthetic efficiency, and the ability to thrive in diverse environments (Mahata et al. 2021; Sarkar et al. 2024; US Department of Energy 2024). These microorganisms hold great promise for addressing global challenges by serving as a rich source of protein and bio‐based compounds, while also enhancing energy security through diversified production pathways (Mahata et al. 2022; Mahata, Dhar, et al. 2023; Zhang et al. 2025). Additionally, microalgae are increasingly explored for their applications in pharmaceuticals, cosmetics, and animal feed, and their integration into advanced biorefinery approaches enables the coproduction of diverse by‐products such as amino acids, lipids, pigments, polysaccharides, vitamins, and minerals, further underscoring their economic and ecological importance (Kumar et al. 2020; Lee et al. 2022; Mahata, Mishra, et al. 2023).

Despite their promise, outdoor pond production of microalgae faces several challenges (Davis et al. 2024). Variability in environmental conditions, such as light availability, temperature fluctuations, and nutrient levels, significantly affects algal growth rates and biomass composition (Huesemann et al. 2024). The variability in light and temperature is expected to intensify due to an increase in the frequency and severity of extreme weather events (Sun et al. 2024; Yan, Duan, et al. 2025; Yan, Sun, et al. 2025). For example, the Texas cold snap in February 2021 highlighted the vulnerability of outdoor cultivation systems to abrupt and severe weather anomalies. Typically, February in Texas experiences average temperatures ranging from 10°C to 16°C, but during the cold snap, temperatures plummeted to as low as –19°C at Dallas/Fort Worth International Airport (Cohen et al. 2021; Hsu et al. 2022). This drastic deviation from the norm turned what is usually a relatively warm month into an abnormally cold one, with profound implications for outdoor systems reliant on stable environmental conditions. Such events underscore the need for adaptive strategies to mitigate the risks posed by weather variability and ensure the resilience of microalgal production systems (Doss‐Gollin et al. 2021).

The development of biomass forecasting systems have emerged as a useful tool to address these challenges by enabling weather‐informed decision‐making in algal production (Yan et al. 2021, 2023; Gao et al. 2022). Unlike most previous scenario‐based studies on biomass growth prediction, which focus on long‐term average biomass production under fixed operating conditions (Sun et al. 2020), biomass forecasting systems aim to bridge the gap between recent advances in biomass growth modeling and data assimilation algorithms (Yan and Moradkhani 2016; Ahmadalipour et al. 2017; Yan et al. 2018), and the needs of practical, operational decision‐making. Short‐term (i.e., weekly) algal forecasting systems have been developed and tested primarily for two applications: (1) improving biomass harvesting planning activities, such as labor, equipment, storage, and downstream logistics, by providing timely biomass production forecasts, including the best harvesting time with adequate preparation (Yan et al. 2021, 2023), and (2) optimizing routine pond operations, specifically the optimal selection of dilution rates (the ratio of harvested media volume to total pond volume), to achieve maximum production potential, such as scheduling a high harvest rate before colder weather sets in (Gao et al. 2022; Yan, Wigmosta, et al. 2025). These studies have demonstrated the potential to bridge gaps in operational planning and provide real‐time insights into the dynamic interactions between algal cultures and their environments.

While short‐term forecasting systems address immediate operational needs, a significant research gap remains in developing tools for longer‐term planning (i.e., monthly to seasonal). Cultivation strategies, such as the selection of algal strain types and pond water depth, are critical for sustainable biomass production. For example, Sun et al. (2020) identified the use of warm or cold algal strains for each month across 5832 locations in the Contiguous United States (CONUS). Their findings suggested that implementing a strain rotation strategy on a monthly basis could significantly enhance biomass yields. They also demonstrated that strain selection varies by location and season; for instance, warm strains are suitable even for winter in Florida, while the choice between cold and warm strains becomes less straightforward during spring to early summer. Moreover, even within a season, selecting among multiple cold or warm strains and pairing them with the optimal pond water depth presents additional challenges for maximizing production (Sun et al. 2020; Xu et al. 2020).

**Figure 1:**
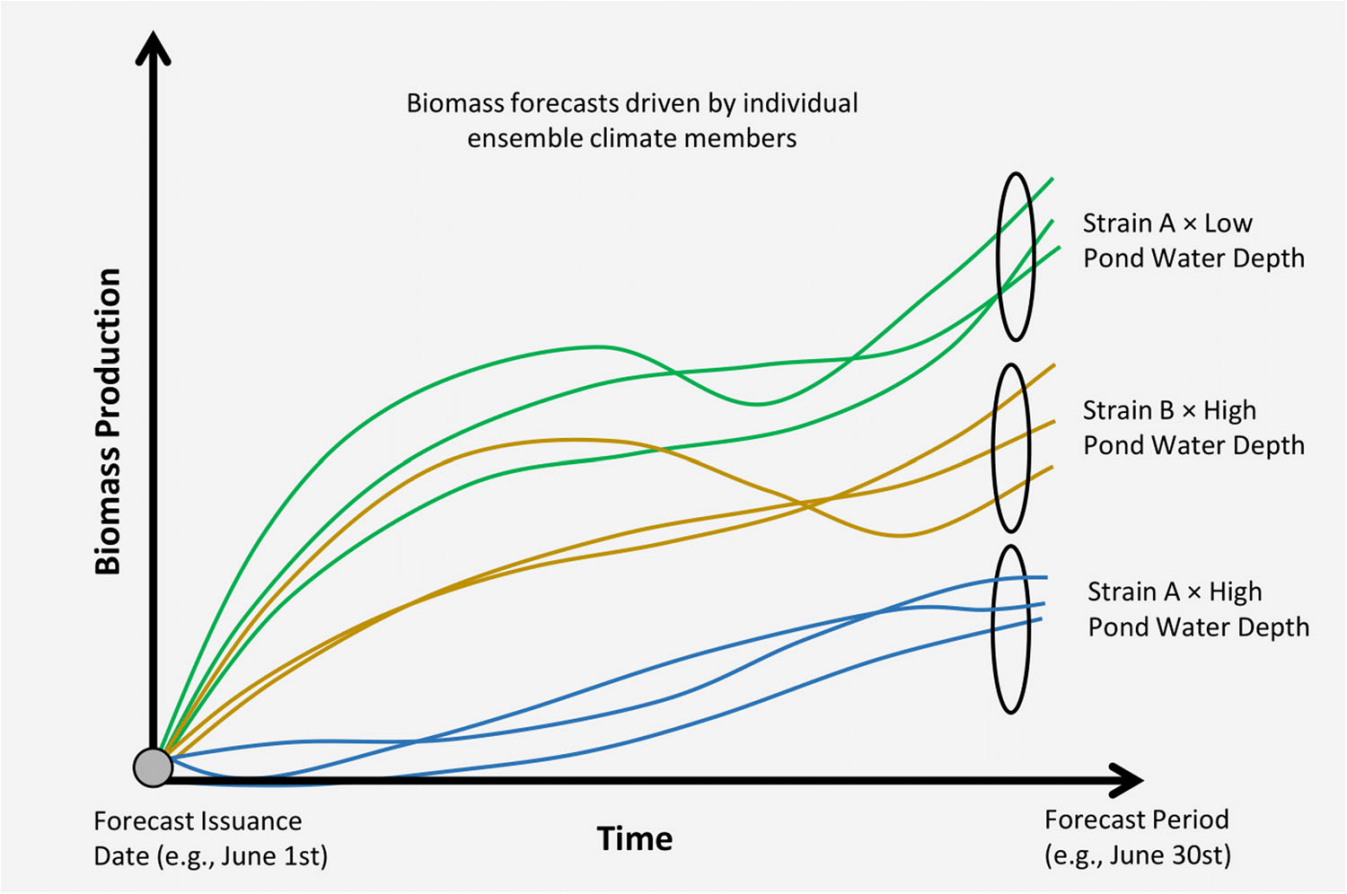
A schematic of the monthly algal forecasting system used to predict biomass production for two algal strains (A, B) at two pond water depths (low and high). The highest predicted production, achieved with strain A at a low pond water depth, is selected as the cultivation strategy to guide the next month's algal growth.

Currently, there is a lack of systems to guide these critical decisions, such as determining the best algal strain and optimizing pond water depth based on projected weather conditions. To address this gap, here the authors are building upon the foundation of short‐term biomass forecasting to develop an experimental monthly forecasting system. This system aims to provide a broader planning horizon, enabling producers to take a proactive and informed approach to managing their cultivation systems (Figure 1). Specifically, the authors couple the Pacific Northwest National Laboratory's (PNNL's) Biomass Assessment Tool (BAT) (Gao et al. 2023; Davis et al. 2024; Yan, Wigmosta, et al. 2025) with two types of monthly forecast data to test the system's accuracy in determining optimal cultivation strategies. The first weather forecast source is the operational seasonal forecast data from the North American Multi‐Model Ensemble (NMME), which consists of multiple coupled models from North American modeling centers (Becker et al. 2022). The second source is climatological forecasts, which use data from Phase 2 of the North American Land Data Assimilation System (NLDAS‐2) (Xia et al. 2012b). These forecasts are constructed by compiling weather data for the same calendar month across all available historical years. For example, to produce a climatological forecast for January 2023, the authors use NLDAS‐2 data from all previous January (e.g., 1979 to 2022). This historical ensemble represents the typical range of conditions expected for that month, allowing us to estimate biomass outcomes under average seasonal patterns without relying on real‐time weather predictions.

This forecasting framework is useful in its ability to integrate monthly predictions into operational planning, offering flexibility to adapt to a wider range of high‐performing strains and diverse environmental conditions. It also enables exploration of different strain–depth combinations to meet specific production objectives beyond simple yield maximization. By providing a systematic, adaptive approach to decision‐making, this study lays the foundation for future field validation, incorporation of additional strains, long‐term seasonal forecasts, and application across geographically diverse sites. The rest of the paper is organized as follows: Section 2 describes the methods and data sources, which includes the models within the BAT, the microorganisms and media, and weather forecast data. Section 3 presents the performance of the experimental monthly algal forecasting system in guiding cultivation strategies. Finally, Section 4 concludes the paper and provides future research directions to advance the algal forecasting system.

## 2 Methods and Data Sources

### 2.1 Experimental Design

**Figure 2:**
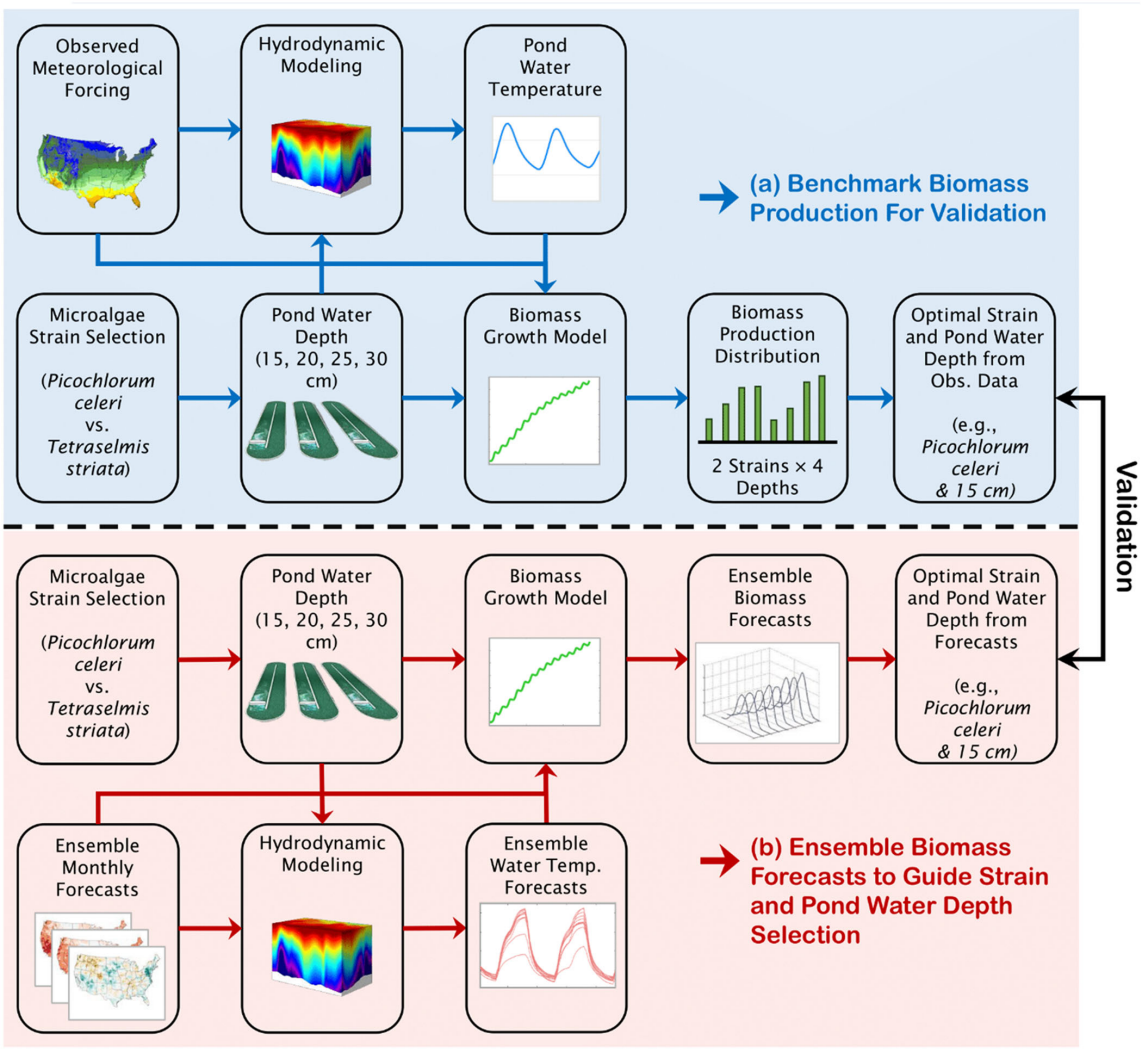
Flowchart of the experimental design demonstrating the use of in‐situ observed meteorological data to validate the application of ensemble monthly forecasts (NMME and NLDAS‐2 climatological forecasts) in predicting biomass production and guiding the selection of algal strain type and pond water depth. NLDAS‐2, Phase 2 of the North American Land Data Assimilation System; NMME, North‐American Multi‐Model Ensemble.

Figure 2 presents the experimental design flowchart used to validate the monthly biomass forecasting system. The process began by using in‐situ observed meteorological data to drive both the calibrated hydrodynamic and biomass growth models, generating monthly biomass production distributions. These simulations enabled the identification of the optimal cultivation strategy, defined by the strain type and pond water depth, for maximizing biomass production. In this proof‐of‐concept study, the authors considered two algal strains: a warm‐temperature strain (*Picochlorum celeri*, hereafter *P. celeri*) and a cold‐temperature strain (*Tetraselmis striata*, hereafter *T. striata*), along with four pond water depths (15, 20, 25, and 30 cm), resulting in eight possible strain–depth combinations. For each month across several validation years (January 2020 to June 2024), the outputs from these simulations were treated as synthetic ground truth due to the lack of long‐term field data across multiple strains and depths (Figure 2a). Relying on model simulations driven by observed weather data as the benchmark was necessary under these circumstances. The validation period was selected based on the availability of NMME multi‐model forecast data.

Next, weather forecast data were used to drive the same models, generating monthly biomass forecasts. For each month, ensemble biomass forecasts were produced for all strain–depth combinations. The ensemble mean was then used to identify the strategy yielding the highest biomass production. These forecast‐informed strategies were compared against the synthetic ground truth to assess the forecasting system's ability to guide optimal cultivation decisions (Figure 2b). Accuracy was evaluated using the confusion matrix method (Stehman 1997). Following the accuracy assessment, the authors compared the biomass production informed by the forecasting system with the production achieved under the current operational strategy used by the Arizona Center for Algae Technology and Innovation (AzCATI) during the Fiscal Year (FY) 2024 State of Technology (SOT) campaigns. All comparisons were based on simulation results to ensure a fair and consistent evaluation. In the SOT strategy, a fixed pond water depth of 20 cm was used year‐round, with *T. striata* cultivated from November to March and *P. celeri* used during the remaining months. As the biomass growth model does not consider contamination, invasive species, or other real‐world constraints, and instead assumes ideal growing conditions, the simulated biomass values are expected to exceed those observed in the field. By holding all other factors constant and varying only the cultivation strategy (strain type and pond water depth), any increase in biomass production relative to the SOT baseline can be attributed to the effectiveness of the forecasting system in guiding optimal cultivation decisions.

### 2.2 Biomass Assessment Tool (BAT)

The PNNL's BAT is an integrated platform designed for modeling, analysis, and data management to assess national‐scale resource and algal biomass production potential in both open pond and closed system facilities. Operating at a high spatial and temporal resolution (e.g., 30–500 m and hourly time step) across the CONUS, the BAT integrates various models and analyses. As detailed in previous studies (Wigmosta et al. 2011; Coleman et al. 2014; Xu et al. 2020), the BAT includes (1) a microalgae growth model, (2) a two‐dimensional hydrodynamic mass and energy balance model, (3) a multiscale land suitability model, (4) tradeoff analysis tools for evaluating biomass production potential with available land and water resources, (5) water source and use intensity analysis, (6) models for nutrient and CO_2_ flue gas sources, availability, and demand, (7) least‐cost transport models for water, nutrients, CO_2_, and refinery access, (8) a land valuation and acquisition model, and (9) a site leveling model.

In this forecasting experiment, the authors used only the microalgae growth model, Huesemann Algae Biomass Growth Model (BGM) (Huesemann et al. 2016), and the hydrodynamic model, Modular Aquatic Simulation System in Two Dimensions (MASS2, version 0.27) (Perkins and Richmond 2004), within the BAT. The mathematical descriptions of both BGM and MASS2 have been extensively covered in prior literature (Venteris et al. 2014; Huesemann et al. 2017; Gao et al. 2018; Khawam et al. 2019; Yan et al. 2023; Yan, Wigmosta, et al. 2025); thus, only a brief summary is provided here. For further details, the authors refer to Huesemann et al. (2016) for BGM and Perkins and Richmond (2004) for MASS2.

The BGM assumes optimal pH, nutrient‐replete, and well‐mixed operating conditions without invasive species. It uses incident light intensity (i.e., photosynthetically active radiation [PAR]) and pond water temperature to determine the biomass growth rate. For each algae strain, the specific growth rate, including the loss rate at night through dark respiration, is experimentally measured in laboratory cultures as a function of light and water temperature. MASS2 is an unsteady flow, two‐dimensional model that simulates hydrodynamics and water quality in ponds, rivers, and estuaries. It uses a structured multiblock, curvilinear computational mesh to discretize the outdoor pond volume and a finite volume method to solve the energy conservation equation. The meteorological inputs for MASS2 include air temperature, dew point temperature, wind speed, air pressure, and shortwave radiation. MASS2 parameters (e.g., conduction rate) have been calibrated to represent elevated outdoor pond conditions and have been shown to match observed pond water temperature data in the field well, as demonstrated by Yan et al. (2023).

For each monthly forecast, both MASS2 and BGM are initialized from the same starting condition (i.e., an initial algal concentration of 0.05 g/L for BGM and an initial pond water temperature of 5°C for MASS2) to eliminate the influence of the initial condition uncertainty on the results. A threshold of 0.5 g/L is set to trigger harvesting. Following each harvest, a residual concentration of 0.1 g/L is maintained in the pond to support continued growth. At the end of each month, the harvested biomass and the remaining algae in the pond are combined to calculate the total biomass production for that month.

### 2.3 Microorganisms and Media

The two microalgae strains used for this modeling practice were *P. celeri* TG2 (warm season strain) and *T. striata* LANL1001 (cold seasons strain). Both strains were the current top‐performing strains in the DISCOVR (Development of Integrated Screening, Cultivar Optimization, and Verification Research) consortium project, selected via rigorous indoor and outdoor evaluations (Gao et al. 2023; Huesemann, Edmundson, et al. 2023; Huesemann, Gao, et al. 2023, 2024). The two strains have demonstrated high biomass productivity and robust growth in outdoor pond trials (McGowen et al. 2023). To model biomass growth under varying climate conditions, a quantitative relationship was established between specific growth rate and light intensity at a given water temperature for the two strains. The parameterization was conducted in a bench‐scale photobioreactor (Multi‐Cultivator MC1000, Photon Systems Instruments, Czech Republic). The MC1000 consisted of 8 columns, each with a working volume of 80 mL. Light intensity in each column can be individually controlled between 0 and 2000 μmol/m^2^/s. Light cycle was set to 12‐h light: 12‐h dark. Water temperature was regulated via an external circulating bath (Polyscience, Illinois, USA). The warm season strain *P. celeri* was parameterized at temperatures between 20°C and 35°C, and the cold season strain *T. striata* was parameterized at temperatures between 5°C and 30°C. In all cultures, biomass concentration was maintained between 0.1 and 0.3, measured as optical density at 720 nm (OD_720_), via automated dilution. The change in OD_720_ between successive dilutions was used to determine the specific growth rate for each light intensity and temperature combination. Measurements were repeated over weeks until stable specific growth rates were achieved. In addition, the dark respiration rate was determined during the 12‐h dark cycle by measuring the decrease in biomass concentration, repeatedly daily until consistent results were obtained. CO_2_ was continuously supplied at 0.5% (v/v) to sustain pH near 7 during the experiments. Culture medium used for the parameterization followed the same composition as in the DISCOVR strain screening pipeline, containing 0.2 g/L ammonium sulfate, 0.012 g/L diammonium phosphate, 0.3 g/L sodium bicarbonate, 0.5 mg/L cyanocobalamin, and trace metals (Huesemann, Edmundson, et al. 2023). The salinity for growth simulation was set to 50 PSU (practical salinity unit) for *P. celeri*, and 35 PSU for *T. striata*. Because the biomass growth model was built upon these parameterization results, growth simulations performed in this study assumed pH 7 condition in the medium specified above.

### 2.4 In‐Situ Meteorological Data

In this study, the authors used in‐situ station measurements as the ground truth, which are widely considered the gold standard for model validation. This approach avoids relying on reanalysis or gridded data, which can introduce inherent uncertainties, particularly at local scales. The in‐situ meteorological data used for the validation period, spanning from January 1, 2020 to June 30, 2024, include hourly records of air temperature, precipitation, dew point temperature, relative humidity, wind speed, and air pressure. These data were obtained from the Arizona Automated Surface Observing System (ASOS) station at Mesa/Falcon Field, located at Mesa Gateway Airport. However, this station does not report solar radiation. To address this gap, the authors obtained hourly solar radiation data from the nearest available in‐situ station, Phoenix Encanto, which is part of the Arizona Meteorological Network. Links to download data from both stations are provided in the Data Availability Statement section.

### 2.5 Ensemble Weather Forecasts

#### 2.5.1 NMME Forecasts

The NMME is a seasonal forecasting system that integrates predictions from multiple coupled atmosphere‐ocean general circulation models (GCMs) developed by U.S. and Canadian climate research centers (Becker et al. 2022). Models included in the NMME suite, such as CCSM4 (Community Climate System Model version 4), CFSv2 (Climate Forecast Model version 2), GEOS‐5 (Goddard Earth Observing System Model version 5), Canadian Coupled Climate Model versions 4 (CanCM4), and GEM/NEMO (Global Environmental Multiscale/Nucleus for European Modeling of the Ocean), provide ensemble forecasts of meteorological variables like temperature and precipitation up to 12 months in advance. NMME data are available at daily or 6‐hourly intervals with a 1° × 1° spatial resolution, and most datasets offer 10 ensemble members per variable. The system is updated monthly. Previous studies have demonstrated NMME's skill in forecasting seasonal hydrometeorological anomalies with reasonable accuracy over North America and other regions (Kirtman et al. 2014; Shukla et al. 2019; Slater et al. 2019).

**Figure 3:**
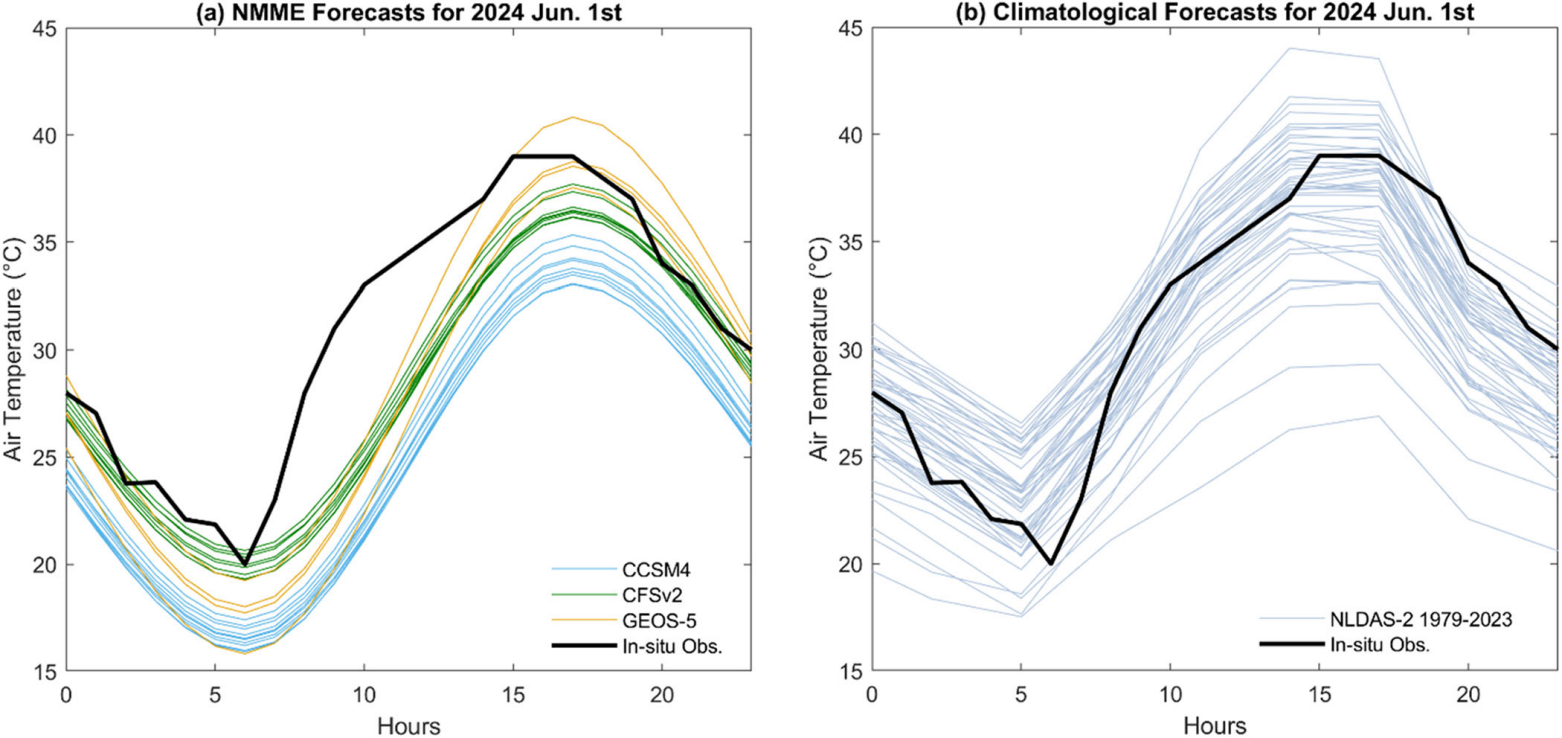
Examples of (a) NMME and (b) NLDAS‐2 climatological ensemble hourly air temperature forecasts for June 1, 2024. NMME hourly air temperature is derived from daily maximum and minimum air temperature data using the MTCLIM algorithm. The NLDAS‐2 climatological hourly air temperature is constructed using historical data from all June 1 records between 1979 and 2023. The black line represents the in‐situ observed air temperature on June 1, 2024, from the Mesa/Falcon Field station. NLDAS‐2, Phase 2 of the North American Land Data Assimilation System; NMME, North‐American Multi‐Model Ensemble.

In this study, the authors obtained daily NMME monthly forecast data, including specific humidity, air pressure, maximum and minimum air temperature, precipitation, and wind speed, from three GCMs (CCSM4, CFSv2, and GEOS), based on data availability from January 2020 through June 2024. Since NMME provides only daily data and lacks solar radiation forecasts, while the BGM requires hourly input and light intensity to simulate microalgae growth during the day and loss during the night, the authors temporally disaggregated the daily NMME forecasts to hourly resolution using the Mountain Microclimate Simulation Model (MTCLIM) (Hungerford et al. 1989; Yan et al. 2020; Sun et al. 2022). MTCLIM estimates sub‐daily air temperatures by applying a third‐order polynomial fit based on the daily temperature range (Figure 3a). Radiation and relative humidity are estimated using the daily temperature range, precipitation, and solar geometry. For each forecast month, CCSM4 provides 10 ensemble members, CFSv2 provides 8 members, and GEOS provides 4 members, resulting in a total of 22 ensemble members. Consequently, MASS2 and BGM are run 22 times for each month for each cultivation strategy. During the validation period from January 2020 to June 2024 (a total of 54 months), data from all three GCMs are available for most months, with only a few months missing: 4 months for CCSM4, 5 months for CFSv2, and 3 months for GEOS.

#### 2.5.2 NLDAS‐2 Climatological Forecasts

NLDAS‐2 provides hourly meteorological forcing data, including precipitation, air temperature, surface pressure, wind speed, specific humidity, incoming solar radiation, and incoming longwave radiation (Xia et al. 2012b). NLDAS‐2 was developed based on the first phase of the NLDAS project, which was initiated to generate reliable initial land surface states for coupled atmosphere‐land models and improve weather predictions. Most of the NLDAS atmospheric forcing data are derived from the North American Regional Reanalysis (NARR), which features a 32‐km spatial resolution and a 3‐h temporal resolution. The NLDAS software interpolates the coarse‐resolution NARR data to a finer spatial scale of 1/8° (~12 km) on the NLDAS grid cell and a temporal resolution of 1 h. NLDAS‐2 archives the forcing data set from 1979 to the present with a 3‐day lag. The NLDAS‐2 data have undergone extensive quality control and validation (Xia et al. 2012b, 2012a, 2015). The assimilated data are derived from the best available real‐time and retrospective in situ and remotely sensed observations of precipitation and shortwave radiation, as well as model‐based forcing data for additional meteorological variables. They also account for interactions between variables. For this study, the authors acquired NLDAS‐2 meteorological data from the 1/8° grid cell closest to Mesa, Arizona, spanning the period from January 1, 1979, to June 30, 2024.

To generate climatological forecasts, the authors adopted an ensemble approach similar to the Ensemble Streamflow Prediction (ESP) method commonly used in flood and drought forecasting (Day 1985; Yan et al. 2017; Zarekarizi et al. 2021). For each forecast month, the authors utilized all historical NLDAS‐2 forcing data available for that specific month across prior years to construct an ensemble of plausible future scenarios. For example, the forecast for June 2023 was based on June forcing data from 1979 to 2022, while the forecast for June 2024 incorporated data from 1979 to 2023 (Figure 3b). This approach assumes the historical variability of climate conditions provides a representative sample of possible future outcomes, enabling skillful probabilistic forecasts in the absence of dynamic climate model predictions. The climatological forecasts were updated annually as new data became available. As a result, the authors generated 41 ensemble forecasts for each month in 2020 and 45 in 2024, leading to 41 and 45 runs of MASS2 and BGM per month for each cultivation strategy, respectively.

### 2.6 Variance Analysis and Evaluation Metrics

In this study, the authors first used BGM simulations driven by historical NLDAS‐2 meteorological data from 1979 to 2023 to evaluate the interannual variability of biomass production and to examine how microalgal strain type, pond water depth, and their interaction influence biomass yields. To quantify these effects, the authors conducted a two‐way analysis of variance (ANOVA), a statistical approach that partitions the total variance in biomass production into components attributable to each main factor (strain type and water depth) and their interaction.

Let $Y_{\mathrm{ijk}}$ represent the biomass production in a specific month for the i‐th strain type (i = 1, 2, e.g., *P. celeri* and *T. striata*), the j‐th pond water depth (j = 1, 2, 3, 4, corresponding to depths of 15, 20, 25, and 30 cm), and the k‐th year (k = 1, 2, …, 45, corresponding to years from 1979 to 2023). The two‐way ANOVA model is given by:

(1) $$Y_{\mathrm{ijk}}=\mu +\alpha_{i}+\beta_{j}+{\left(\alpha \beta \right)}_{\mathrm{ij}}+\varepsilon_{\mathrm{ijk}}$$

where μ is the overall mean biomass production, $\alpha_{i}$ is the effect of strain type i, $\beta_{j}$ is the effect of pond water depth j, ${\left(\alpha \beta \right)}_{\mathrm{ij}}$ is the interaction effect between strain type i and pond water depth j, and $\varepsilon_{\mathrm{ijk}}$ is the random error term, assumed to be independently and identically distributed with mean 0 and constant variance.

For each month, the authors calculated the proportion of total variance explained by each component, strain type, pond depth, and their interaction, by computing the ratio of the corresponding sum of squares to the total sum of squares. These variance ratios range from 0 to 1, where a higher value indicates that the factor contributes more substantially to the variability in biomass production. Additionally, to assess the consistency of cultivation strategies over time, the authors determined the occurrence probability of each strategy (defined by strain type and pond depth) being the optimal one for maximizing biomass production. For each month, this probability was calculated as the fraction of years (1979–2023) in which a given strategy produced the highest biomass. The occurrence probability also ranges from 0 to 1. A higher value indicates greater robustness of the strategy to interannual variability, with a value of 1 signifying that the same strategy yielded the highest biomass in all years for that month.

The forecasting skill of both NMME and climatological predictions for air temperature and precipitation is assessed using the probabilistic metric known as the Continuous Ranked Probability Score (CRPS) (Hersbach 2000; Sun et al. 2019), defined as:

(2) $$\mathrm{CRPS}=\int {\left[F\left({\hat{x}}_{t}\right)-H({\hat{x}}_{t}\geq x_{t})\right]}^{2}\;d{\hat{x}}_{t}$$

where ${\hat{x}}_{t}$ denotes the forecast value, $x_{t}$ is the corresponding observed value, $F\left({\hat{x}}_{t}\right)$ represents the cumulative distribution function (CDF) of the forecast, and H(∙) is the Heaviside step function. The CRPS ranges from 0 to ∞, with lower values indicating better forecast performance. It simultaneously accounts for both the accuracy of the forecast and the spread of uncertainty. A CRPS value closer to 0 implies higher forecast skill and generally smaller uncertainty.

To assess the accuracy of forecast‐informed cultivation strategies, a confusion matrix (Stehman 1997) was constructed by comparing the forecast‐based strategy selection against the observation‐based optimal strategy for each month from January 2020 to June 2024. Each cell in the matrix represents the number of months a specific combination of forecasted and actual strategies occurred. A higher concentration of values along the diagonal indicates better agreement between forecasts and observations. Forecast accuracy is then calculated as the proportion of correctly predicted strategies, represented by the sum of the diagonal elements, relative to the total number of months evaluated.

## 3 Results and Discussion

### 3.1 Variance of Monthly Biomass Production

**Figure 4:**
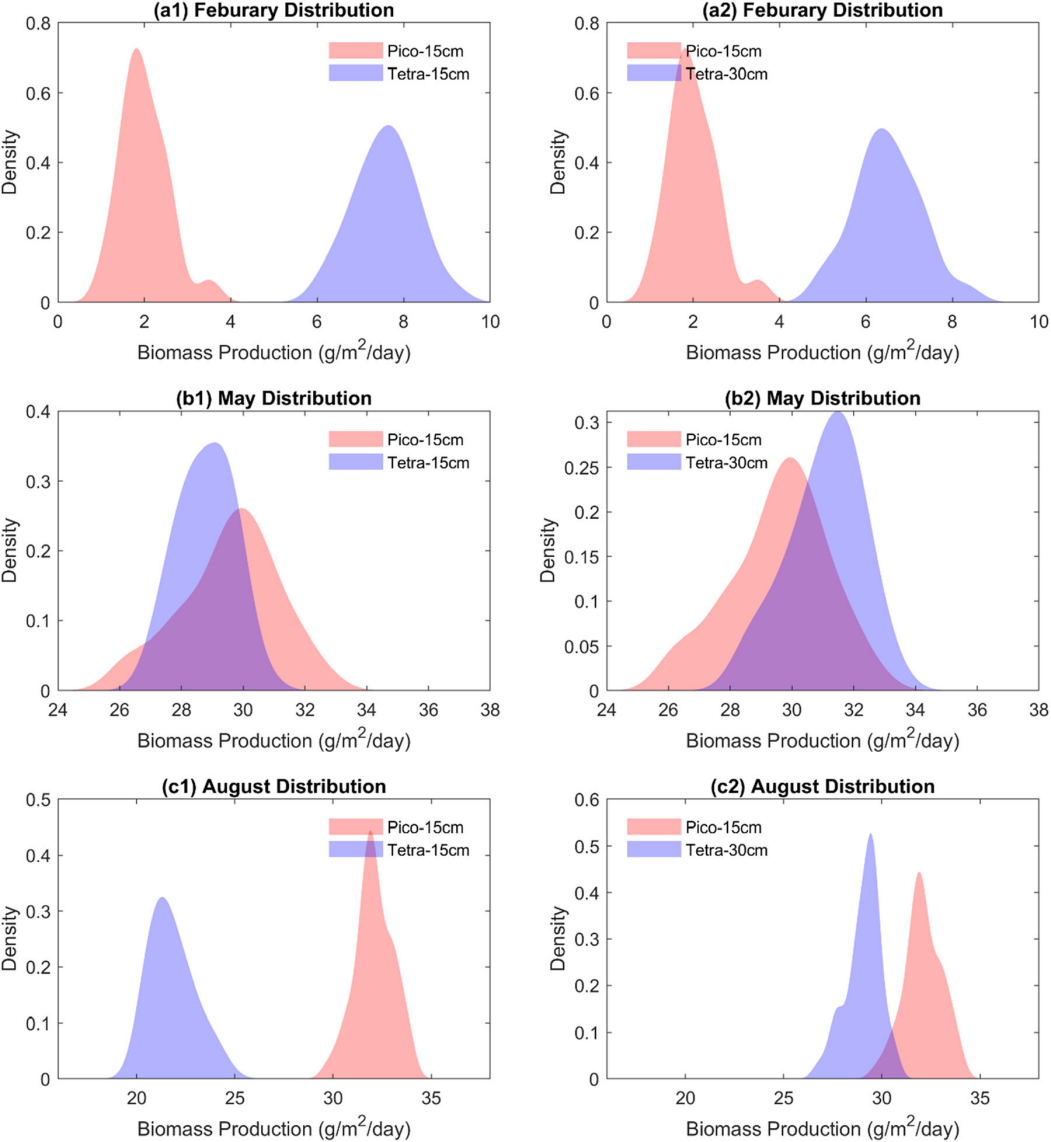
Probability density function (PDF) of biomass production for February, May, and August from 1979 to 2023. Biomass production exhibits greater variance due to interannual climate variability. Two pond water depths are included to illustrate the impacts of strain type and pond water depth selection on biomass production.

Figure 4 presents the distribution of biomass production for the months of February, May, and August over the period from 1979 to 2023. The results highlight the critical role of both strain and pond water depth selection in enhancing biomass yield. During the winter month of February, *T. striata* consistently outperforms *P. celeri*, producing higher biomass at both 15 and 30 cm pond water depths compared to *P. celeri* at 15 cm. In contrast, during the summer month of August, *P. celeri* grown at a 15 cm pond water depth surpasses *T. striata* in biomass production at the same depth and generally outperforms *T. striata* at 30 cm as well. This seasonal reversal is consistent with the higher temperature tolerance and light utilization efficiency of *P. celeri*, which performs better under warm, high‐radiation conditions (Gao et al. 2023). The most intriguing pattern emerges in May, a transitional season. Here, *P. celeri* at a 15 cm pond water depth tends to outperform *T. striata* at the same water depth in terms of mean biomass production but exhibits greater variability. In contrast, *T. striata* at 30 cm often shows a higher probability of yielding greater biomass than *P. celeri* at 15 cm. This suggests that the interaction between strain type and pond water depth becomes especially important during transitional periods. Such interactions likely reflect shifts in light penetration and thermal stratification, which affect growth dynamics differently across strains (Huesemann et al. 2016). Additionally, Figure 4 reveals the significant influence of interannual climate variability on biomass production. For example, the coefficient of variation (CV) in February for *T. striata* at 15 cm depth is 0.09, indicating a 9% relative variability around the mean production rate. This level of variability should be considered when evaluating long‐term productivity and operational strategies.

**Figure 5:**
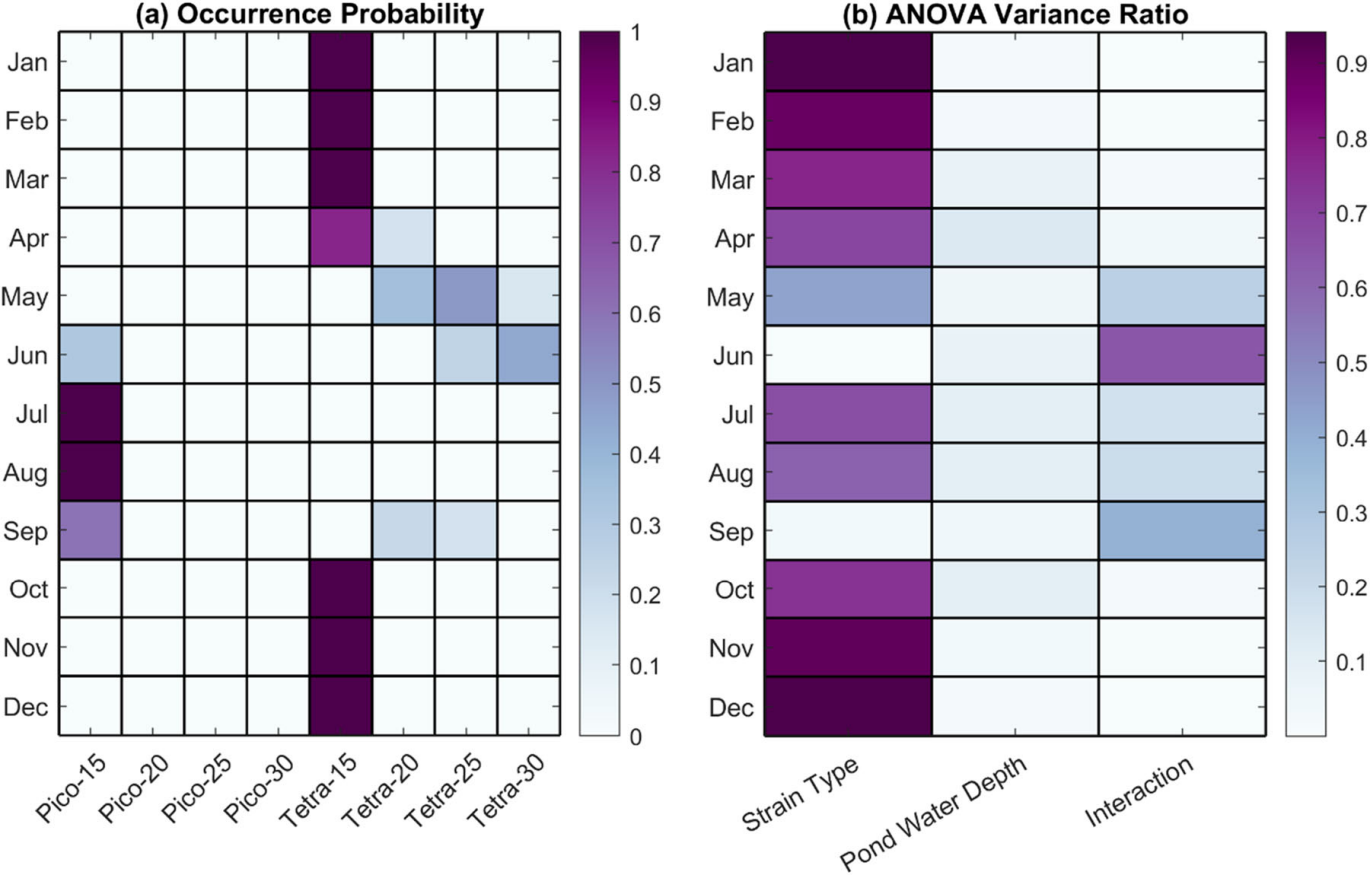
(a) The occurrence probability of each cultivation strategy (strain type + pond water depth) for each month from 1979 to 2023. (b) Ratio of variance explained by each factor (strain type, pond water depth, and their interaction) to the total variance in biomass production, as determined by a two‐way ANOVA from 1979 to 2023. Larger values indicate a greater effect of the factor on biomass production variability. ANOVA, analysis of variance.

Figure 5a illustrates the monthly occurrence probability of each cultivation strategy over the period from 1979 to 2023. The results reveal clear seasonal preferences for specific strain and pond depth combinations. *T. striata* at 15 cm pond depth demonstrates a high probability of selection, close to or equal to 1, from October through March, suggesting it is the most favorable option during the cooler months. In contrast, *P. celeri* at 15 cm shows a higher likelihood of selection during the summer months, with occurrence probabilities exceeding 0.6 from July to September. In April, *T. striata* at 15 cm remains the most likely strategy, though the 20 cm depth option also gains moderate support, with a probability around 0.2. For May, *T. striata* continues to be preferred, but the highest probability shifts to deeper pond depths, specifically 20 cm and 25 cm, indicating changing environmental conditions that favor these configurations. The situation becomes more complex in June, where the model shows moderate probabilities for *P. celeri* at 15 cm and *T. striata* at 30 cm, suggesting no clear dominant strategy for that month and a possible need for adaptive decision‐making. Figure 5b presents the results of an ANOVA analysis, showing the variance ratio associated with three factors: strain type, pond water depth, and their interaction. The analysis indicates that strain type is the dominant source of variance in both winter and summer, meaning that the choice of algal strain primarily drives biomass production during those seasons. However, during the transition months, including May, June, and September, the interaction between strain type and pond depth plays a more substantial role. In particular, for June and September, the interaction accounts for 64% and 39% of the total variance in biomass production, respectively. This highlights the importance of jointly considering both factors when optimizing cultivation strategies under variable seasonal conditions.

These findings underscore the importance of incorporating forecasting tools into cultivation planning. Due to significant interannual climate variability, selecting the optimal combination of algal strain and pond water depth is not always straightforward, especially during transition seasons such as May, June, and September. In these months, both *T. striata* and *P. celeri* may exhibit favorable growth potential depending on environmental conditions, and the effectiveness of different pond depths can shift accordingly. A monthly biomass forecasting system could help anticipate growing conditions, allowing for more informed and dynamic selection of cultivation strategies. For example, in years expected to be cooler or cloudier than average, early‐season forecasts could signal a better fit for *T. striata* at greater pond depths, whereas warmer projections might favor *P. celeri* in shallower ponds.

### 3.2 Weather Forecast Evaluation

**Figure 6:**
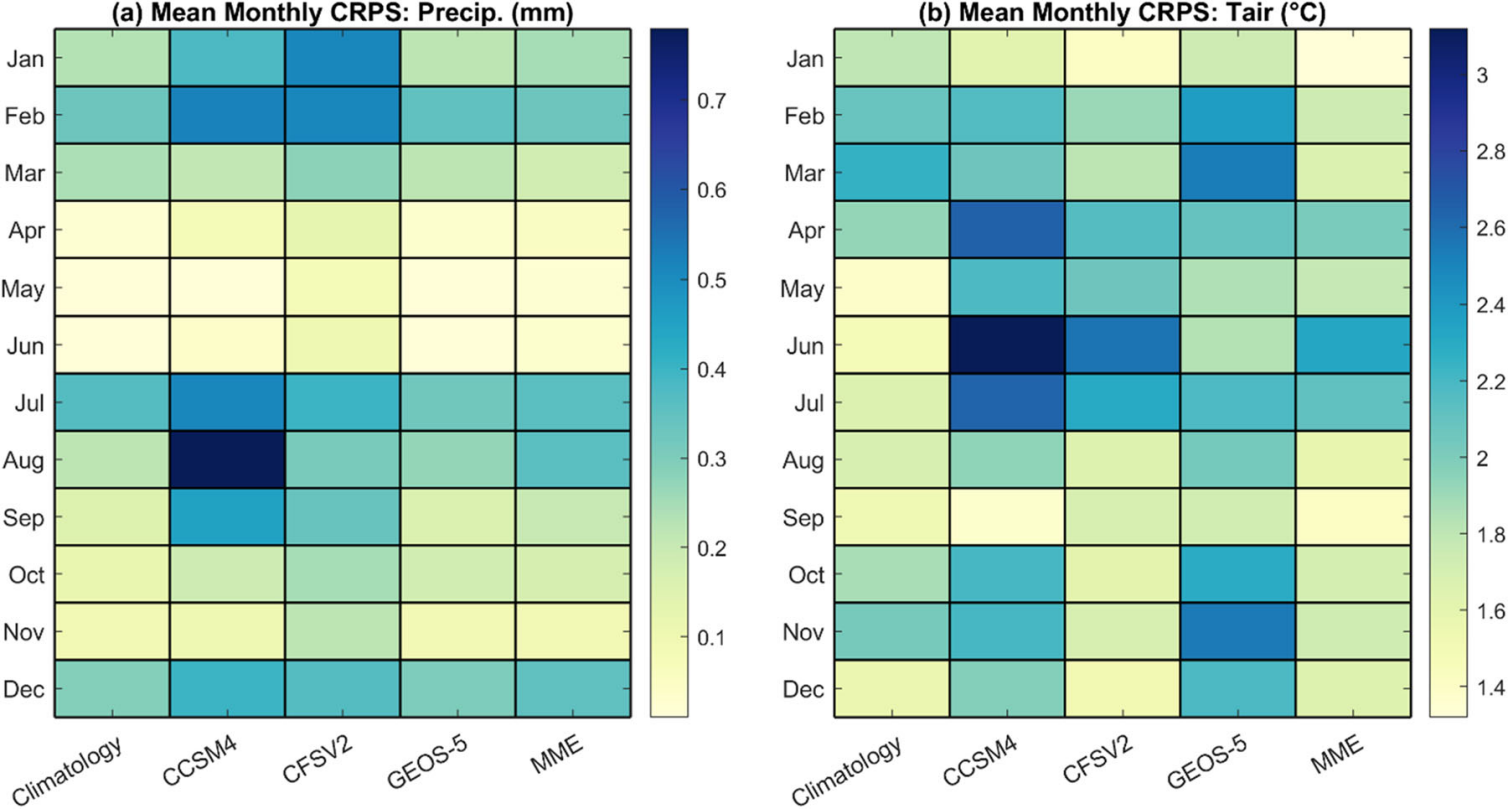
Mean monthly CRPS values for (a) precipitation and (b) air temperature from January to December, comparing the NLDAS‐2 climatological forecast with each NMME individual model and the multi‐model ensemble (MME). The evaluation period spans January 2020 to June 2024. NLDAS‐2, Phase 2 of the North American Land Data Assimilation System; NMME, North‐American Multi‐Model Ensemble.

Figure 6 presents the mean monthly CRPS values for daily precipitation and air temperature forecasts, providing an assessment of forecast accuracy across different months. Weather forecast performance was evaluated using data from 2020 to 2024, covering seasonal variability at the Mesa, Arizona testbed. The CRPS values are calculated by comparing the forecasts to in‐situ observations. For precipitation, both the NLDAS‐2 climatological and NMME forecasts exhibit higher CRPS values during the winter months (December–March) and the summer months (July–August). This pattern corresponds to the seasonal precipitation regimes in the region: winter storms are predominantly driven by Pacific frontal systems, while summer precipitation is largely associated with the North American Monsoon, which transports moist air from the Gulf of America and the eastern Pacific into the southwestern US (Adams and Comrie 1997). These regimes strongly influence forecast skill because models often misrepresent convective processes during monsoon conditions (Wallace and Minder 2024). During the shoulder seasons (spring and fall), CRPS values for precipitation forecasts tend to be lower, indicating relatively improved forecast skill. On average, the NLDAS‐2 climatological forecast, which is based on historical ensemble, exhibits the lowest CRPS values for precipitation across most months, outperforming the NMME‐based methods. When averaged over all months, the CRPS values are 0.17 mm for the NLDAS‐2 climatological forecast, 0.31 mm for CCSM4, 0.29 mm for CFSv2, 0.18 mm for GEOS‐5, and 0.20 mm for the multi‐model ensemble (MME) approach, which combines forecasts from the three GCMs. However, forecast skill varies by season, and no single method consistently outperforms the others. For example, the MME performs best in March, while the NLDAS‐2 climatological forecast shows superior performance in August.

For air temperature forecasts, the MME provides the best overall performance across all months, with a mean CRPS value of 1.75°C, followed by the NLDAS‐2 climatological forecast at 1.77°C and CFSv2 at 1.86°C. Similar to precipitation, no single method consistently outperforms the others across all months. The NLDAS‐2 climatological forecast performs best during the spring, particularly in May and June, while the NMME models tend to show better performance in the winter months (January through March) and also in September. These differences in forecast accuracy are likely due to the inherent limitations of each approach. The NLDAS‐2 method is based on gridded data that integrates various types of observations but relies on the assumption that historical averages can represent future conditions, an assumption that may not hold under rising temperatures. In contrast, NMME models have coarser spatial resolution and may struggle to capture local climate variability, especially in regions with complex terrain or localized weather patterns. Since the NMME seasonal forecasts do not include solar radiation data, the authors estimated it using the MTCLIM algorithm, which derives solar radiation from other meteorological variables. Understanding how solar radiation is estimated, and the limitations of this approach, is therefore essential.

**Figure 7:**
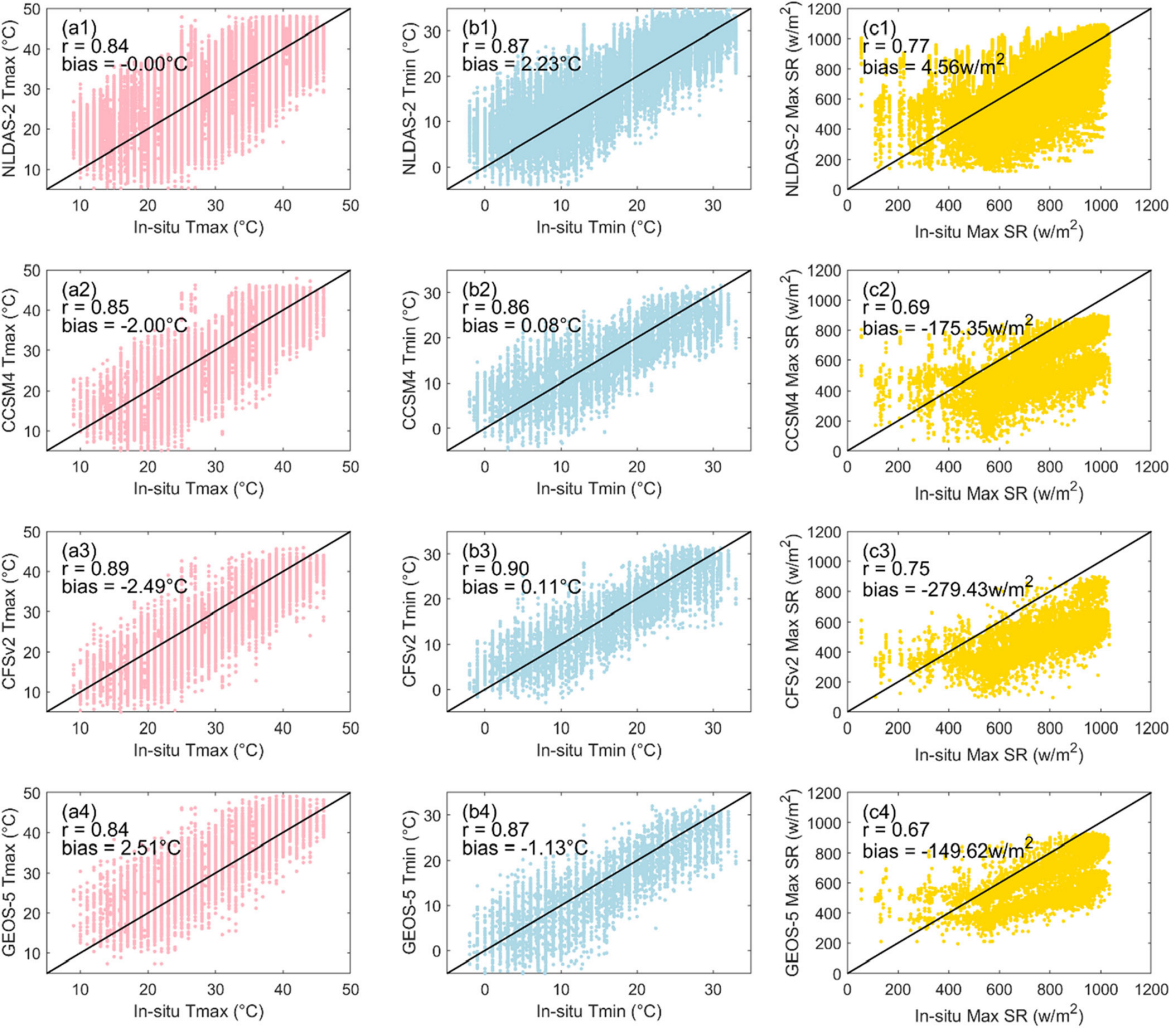
Comparison of NLDAS‐2 climatological and NMME ensemble forecast data for (a1–a4) daily maximum air temperature (Tmax), (b1–b4) daily minimum air temperature (Tmin), and (c1–c4) daily maximum solar radiation (SR) against in‐situ observations during the validation period (January 2020 to June 2024). Pearson correlation coefficients (*r*) and mean bias values are also provided. NLDAS‐2, Phase 2 of the North American Land Data Assimilation System; NMME, North‐American Multi‐Model Ensemble.

Figure 7 compares daily maximum and minimum air temperatures, along with daily maximum solar radiation, from the NLDAS‐2 climatological forecasts and three NMME GCMs (CFSv2, CCSM4, and GEOS‐5) against in‐situ observational data over the validation period. In terms of Pearson correlation, all forecasts show stronger agreement with observations for air temperature (*r* values between 0.8 and 0.9) than for solar radiation (*r* values between 0.67 and 0.75), indicating higher uncertainty in solar radiation forecasts. For air temperature, the NLDAS‐2 climatological forecasts exhibit minimal bias in daily maximum temperature and solar radiation but tend to overestimate daily minimum temperature. The small overall bias in solar radiation masks more significant discrepancies: while the model overestimates low solar radiation values, it substantially underestimates higher observed values, particularly during summer. Among the NMME models, CCSM4 and CFSv2 show similar behavior, underestimating daily maximum temperature and slightly overestimating daily minimum temperature. Both also significantly underestimate solar radiation. In contrast, GEOS‐5 tends to overestimate daily maximum temperature and underestimate daily minimum temperature, with a relatively smaller bias in solar radiation underestimation. Overall, all forecasts perform better for air temperature than for solar radiation. The consistent underestimation of solar radiation across all forecasts, especially during summer months, is a concern. This bias could lead to cooler simulated pond temperatures and, consequently, lower estimates of algal biomass production during periods of peak growth potential. Similar impacts of radiation underestimation on aquatic productivity have been reported in modeling studies of aquaculture and freshwater ecosystems (Hoshovsky et al. 2025).

A likely cause of the solar radiation bias is the substantial error in summer precipitation forecasts observed in both the NMME models and the NLDAS‐2 climatological forecasts, as shown in Figure 6. In the case of the NMME models, this bias is partly due to the way the MTCLIM algorithm infers cloudiness from precipitation occurrence. Excessive precipitation forecasts during the summer lead MTCLIM to estimate increased cloud cover, which in turn reduces modeled solar transmittance and results in lower estimated solar radiation. For the NLDAS‐2 climatological forecast, errors in solar radiation estimation may stem from the high interannual variability associated with the North American Monsoon. This variability affects both the timing and intensity of peak summer rainfall, which influences the derived cloudiness and consequently the estimated solar radiation. To address these limitations, direct solar radiation forecasts can be valuable. Short‐term weather forecast systems, such as the Global Ensemble Forecast System (GEFS), provide direct solar radiation output and have been shown to perform better for this variable (Hamill et al. 2013; Yan et al. 2023). However, GEFS is limited to relatively short forecast horizons (e.g., up to 16 days), whereas NMME is designed for seasonal‐scale prediction. This mismatch in forecast range, combined with the absence of direct solar radiation outputs from NMME and the reliance on empirical estimation methods like MTCLIM, presents a major challenge when using NMME for monthly algal biomass forecasting.

However, it is important to note that the accuracy of daily precipitation, air temperature, and solar radiation forecasts does not necessarily translate directly into the accuracy of algal growth forecasts. The BGM used for biomass forecasting requires hourly inputs, such as water temperature and solar radiation, to simulate pond conditions. The model exhibits highly nonlinear responses to these variables. For instance, if one forecast underestimates water temperature but overestimates solar radiation, the resulting biomass predictions may still be comparable to a forecast that accurately captures both variables, due to compensating effects in the pond's energy balance and algal growth dynamics. Nevertheless, this diagnostic analysis of meteorological forecast accuracy is still useful. It highlights the strengths and limitations of each forecasting method and helps improve understanding of how meteorological uncertainties propagate into algal biomass predictions.

### 3.3 Cultivation Strategy Forecast Evaluation

**Figure 8:**
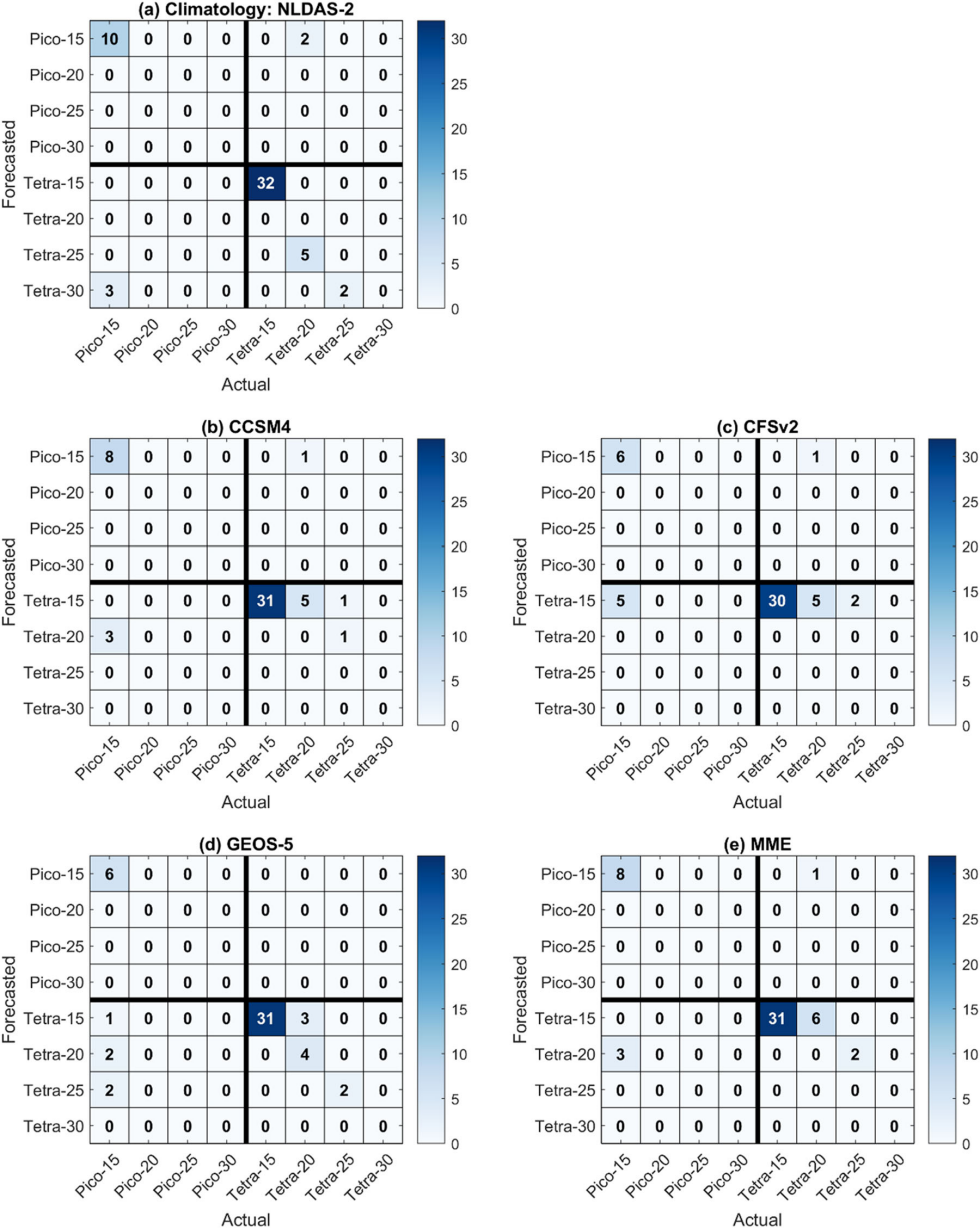
Confusion matrices for the (a) NLDAS‐2 climatological forecast, (b, c, d) three individual NMME models, and (e) the multi‐model ensemble (MME) in predicting the optimal combination of strain type and pond water depth for each month from January 2020 to June 2024. Forecast accuracy is computed as the proportion of correctly predicted strategies, represented by the sum of the diagonal elements, relative to the total number of predictions. NLDAS‐2, Phase 2 of the North American Land Data Assimilation System; NMME, North‐American Multi‐Model Ensemble.

Figure 8 presents the confusion matrices for monthly cultivation strategy selection from January 2020 to June 2024, based on forecasts from the NLDAS‐2 climatology, three individual NMME models (CCSM4, CFSv2, and GEOS‐5), and the MME. The task involves predicting the optimal combination of algal strain and pond water depth for each month, and forecast accuracy is quantified as the proportion of correct predictions relative to the total number of predictions (Table 1). Among the forecasting methods, GEOS‐5 achieved the highest overall accuracy at 84.3%, followed by CCSM4 (78.0%), NLDAS‐2 climatology (77.8%), MME (76.5%), and CFSv2 (73.5%), which showed the weakest performance. The relatively poor accuracy of CFSv2 is consistent with its known systematic biases (Figure 7). As discussed earlier, one of the key sources of forecast error across all models is the cold bias in predicted pond temperatures, primarily driven by the underestimation of incoming solar radiation and daily air temperature. GEOS‐5 stands out in this regard, as it is the only model exhibiting a positive bias in maximum daily temperature and the smallest underestimation in solar radiation, which likely explains its superior performance in strategy prediction.

**Table 1:** Summary of the total forecast accuracy (for both strain type and pond water depth selection) and accuracy for strain type selection only, using the two forecast methods. Evaluation period: January 2020 to June 2024.

Method	Forecast accuracy	
	Total (Strain + Water depth)	Strain only
NLDAS‐2 Climatology	77.8%	90.7%
CCSM4	78.0%	92.0%
CFSV2	73.5%	87.8%
GEOS	84.3%	90.2%
MME	76.5%	92.2%

A deeper look at the confusion matrices reveals that most classification errors stem from incorrect predictions of pond water depth, rather than the strain type. When isolating forecast skill in strain selection alone, all models except CFSv2 exceeded 90% accuracy. Specifically, strain selection accuracies were 90.7% for NLDAS‐2 climatology, 92.0% for CCSM4, 87.8% for CFSv2, 90.2% for GEOS‐5, and 92.2% for the MME. These high accuracies suggest that forecast‐driven strain choice is a relatively robust component of the decision framework, while pond depth selection remains more sensitive to meteorological forecast biases and uncertainties. This robustness likely arises because strain performance is more strongly controlled by broad seasonal patterns, whereas pond depth is highly sensitive to monthly weather variability (Slegers et al. 2013).

**Figure 9:**
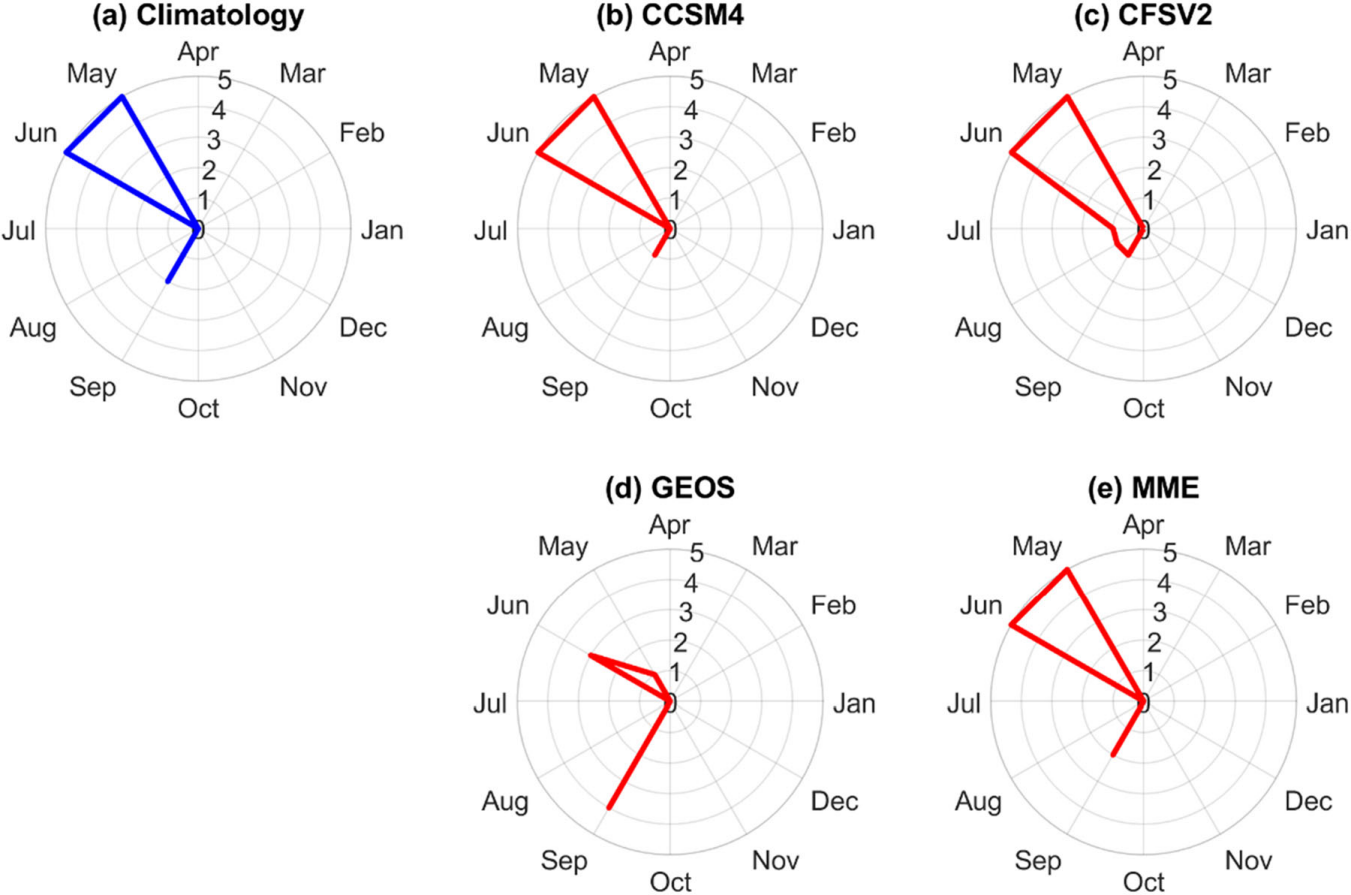
Frequency of months in which the (a) NLDAS‐2 climatology and (b, c, d, e) NMME forecasts indicated a suboptimal cultivation strategy. Evaluation period: January 2020 to June 2024. NLDAS‐2, Phase 2 of the North American Land Data Assimilation System; NMME, North‐American Multi‐Model Ensemble.

Figure 9 highlights the months during which the forecasts led to suboptimal cultivation strategy recommendations. Among all models, including the NLDAS‐2 climatology, the three individual NMME GCMs (CCSM4, CFSv2, GEOS‐5), and the multi‐model ensemble (MME), May, June, and September consistently exhibit the highest error rates. These patterns align with the elevated interannual climate variability observed during these months, as shown in Figure 5, where the ANOVA variance ratios for the interaction between strain type and pond water depth are highest: 0.25 for May, 0.64 for June, and 0.39 for September. For example, the optimal cultivation strategy in May remained consistent from 2020 to 2024, favoring *T. striata* at a 20 cm pond water depth. However, only GEOS‐5 successfully identified the optimal strategy for May in four out of 5 years, while all other methods failed to make a correct selection in any year. In June, the interaction between strain type and pond water depth contributed significantly to uncertainty, with the optimal strategies varying year‐to‐year: *T. striata* at 25 cm in 2020 and 2023, and *P. celeri* at 15 cm in 2021, 2022, and 2024. Again, GEOS‐5 performed best, making three errors, whereas all other methods misclassified the strategy in all 5 years. In September, however, GEOS‐5 showed higher error rates compared to its performance in earlier months. The optimal strategies were *T. striata* at 20 cm (2020), *P. celeri* at 15 cm (2021 and 2022), and *T. striata* at 20 cm (2023). Despite its overall strong performance, GEOS‐5 struggled to capture the correct strategy in September, even though its precipitation forecasts had the lowest CRPS values among the three NMME models (Figure 6). As discussed earlier, lower meteorological forecast error does not always translate to higher decision accuracy, due to the nonlinear and threshold‐based responses in biomass dynamics modeled in the system.

Overall, while GEOS‐5 consistently outperformed the other models in total accuracy, no single forecasting method reliably identified the optimal strategy across all months. The months of May, June, and September emerged as particularly challenging due to their high climate variability and nonlinear interactions in the decision framework. The results emphasize that even models with relatively accurate climate forecasts can still lead to suboptimal decisions if they fail to capture complex environmental conditions that govern algal growth and system performance.

### 3.4 Algae Production Evaluation

To evaluate the practical implications of forecast‐based decision‐making, the authors examined the monthly production losses incurred when the forecasting systems selected suboptimal cultivation strategies. Algal biomass production outcomes under forecast‐informed strategies were analyzed for the 2020–2024 experimental period. Specifically, for each month in which a forecast‐based strategy differed from the optimal strategy (as determined using in‐situ observation data), the authors computed the production difference. Table 2 summarizes the average production losses across the different forecasting methods, relative to the optimal outcome. While the GEOS‐5 forecasting system achieved the highest strategy selection accuracy at 84.3%, its average production loss was 5.4%, not the lowest among the models. In contrast, the NLDAS‐2 climatology‐based forecast had a lower selection accuracy of 77.8%, yet its average production loss was only 2.2%, the smallest among all models. The largest production loss was associated with CFSv2 at 13.4%, followed by CCSM4 at 5.7%. Interestingly, the MME performed better than all three individual NMME models, with an average loss of 4.8%.

**Table 2:** Summary of the average biomass production for the months where the forecast indicates a suboptimal strategy.

Type	Avg. production (g/m^2^/day) for suboptimal months		
	Optimal	Forecast	Diff.
NLDAS‐2 Climatology	30.66	29.97	−2.2%
CCSM4	31.26	29.49	−5.7%
CFSV2	31.60	27.35	−13.4%
GEOS‐5	28.95	27.39	−5.4%
MME	30.71	29.23	−4.8%

These results highlight that selection accuracy alone does not fully capture the practical performance of a forecasting system. The magnitude of error during misclassified months is also critical. For example, most of GEOS‐5's misclassifications occurred in September, when errors tend to have larger impacts on biomass yield. In contrast, NLDAS‐2 climatology method primarily made errors in May and June, when the consequences of a suboptimal choice were less severe. Furthermore, although NLDAS‐2 relies on historical climatology rather than real‐time dynamic forecasts, its strategy recommendations on algae production tend to remain relatively close to the optimal, even during months of high interannual variability. This suggests that, under certain conditions, a simple climatology‐based approach may outperform more sophisticated models, particularly when the latter suffer from large forecast uncertainties. Similar findings have been noted in meteorological forecasting, where climatology sometimes outperforms dynamic models during high‐variability periods (Vogel et al. 2018). In summary, while dynamic weather forecasts (e.g., from NMME models) aim to capture real‐time variability, their associated uncertainty can sometimes lead to strategy selections that result in greater production losses than those based on historical averages. These findings underscore the importance of evaluating both forecast accuracy and the production impact of forecast errors when assessing the value of different forecasting systems for operational decision‐making.

Lastly, the authors compared the forecast‐informed cultivation strategies with the FY2024 SOT baseline strategy. The SOT approach implemented a fixed configuration throughout the year: *T. striata* was cultivated from November to March, *P. celeri* from April to October, and a constant pond water depth of 20 cm was maintained year‐round. To assess potential improvements over this static approach, the authors applied both the NLDAS‐2 climatology‐informed strategy and the GEOS‐5 forecast‐informed strategy, evaluating their performance month by month (summarized in Tables 3 and 4). Note that July and August of 2024 were excluded from this analysis due to missing NMME model data. Both the NLDAS‐2 and GEOS‐5 strategies consistently selected a shallower pond depth of 15 cm in most months, in contrast to the fixed 20 cm depth of the SOT configuration. Moreover, both forecasting approaches favored more frequent use of *T. striata*, particularly during the spring months such as May and June, where it appeared to offer a clear biomass production advantage.

**Table 3:** The NLDAS‐2 climatology‐based forecast‐informed cultivation strategy and production versus the SOT cultivation strategy and production during the fiscal year 2024.

Year	Month	SOT		NLDAS‐2 climatology forecast		Production Diff.
		Strategy (strain‐depth, in cm)	Production (g/m^2^/day)	Strategy (Strain‐Depth, in cm)	Production (g/m^2^/day)	
2023	9	*P. celeri‐20*	21.8	*P. celeri‐15*	23.3	7.0%
2023	10	*P. celeri‐20*	11.9	*T. striata‐15*	18.0	50.9%
2023	11	*T. striata‐20*	10.2	*T. striata‐15*	10.5	3.2%
2023	12	*T. striata‐20*	6.1	*T. striata‐15*	6.6	8.1%
2024	1	*T. striata‐20*	6.0	*T. striata‐15*	6.5	8.5%
2024	2	*T. striata‐20*	10.2	*T. striata‐15*	10.7	4.2%
2024	3	*T. striata‐20*	15.5	*T. striata‐15*	16.0	3.4%
2024	4	*P. celeri‐20*	16.6	*T. striata‐15*	24.6	47.6%
2024	5	*P. celeri‐20*	24.3	*T. striata‐25*	29.5	21.7%
2024	6	*P. celeri‐20*	31.9	*T. striata‐30*	32.0	0.4%
Average			15.45		17.77	15.0%

**Table 4:** The GEOS‐5 forecast‐informed cultivation strategy and production versus the SOT cultivation strategy and production during the fiscal year 2024.

Year	Month	SOT		GEOS‐5 Forecast		Production Diff.
		Strategy (strain‐depth, in cm)	Production (g/m^2^/day)	Strategy (strain‐depth, in cm)	Production (g/m^2^/day)	
2023	9	*P. celeri‐20*	21.8	*T. striata‐15*	24.2	11.0%
2023	10	*P. celeri‐20*	11.9	*T. striata‐15*	18.0	50.9%
2023	11	*T. striata‐20*	10.2	*T. striata‐15*	10.5	3.2%
2023	12	*T. striata‐20*	6.1	*T. striata‐15*	6.6	8.1%
2024	1	*T. striata‐20*	6.0	*T. striata‐15*	6.5	8.5%
2024	2	*T. striata‐20*	10.2	*T. striata‐15*	10.7	4.2%
2024	3	*T. striata‐20*	15.5	*T. striata‐15*	16.0	3.4%
2024	4	*P. celeri‐20*	16.6	*T. striata‐15*	24.6	47.6%
2024	5	*P. celeri‐20*	24.3	*T. striata‐20*	29.9	23.5%
2024	6	*P. celeri‐20*	31.9	*T. striata‐25*	31.4	−1.3%
Average			15.45		17.84	15.5%

One of the most interesting findings was observed in April and October: cultivating *T. striata* at 15 cm pond depth led to a 47.6% and 50.9% increase in biomass production compared to the SOT strategy, which used *P. celeri* at 20 cm. Across all months, the NLDAS‐2 climatology‐informed strategy produced higher biomass yields than the SOT strategy, with monthly gains ranging from 0.4% to 50.9%, and an average increase of 15.0%. Similarly, the GEOS‐5‐informed strategy also outperformed the SOT baseline in every month except June, with gains between 3.2% and 50.9%, and an average increase of 15.5%. It is important to emphasize that all results, including those for the SOT and forecast‐based strategies, are derived from modeling simulations rather than field experiments. The modeling framework assumes idealized growing conditions and does not account for operational challenges such as contamination, pest infestations, equipment failures, or competition from invasive species. As such, while the forecast‐informed strategies show substantial promise in improving biomass yield under modeled conditions, real‐world implementation would require validation and adaptive management to address uncertainties and constraints in practice.

## 4 Conclusions and Future Work

This study demonstrates the feasibility and practical value of a monthly biomass forecasting system to guide algal cultivation strategies, particularly for optimizing strain selection and pond water depth. By integrating the BAT with both climatology‐based (NLDAS‐2) and seasonal dynamical (NMME) forecasts, the system provides a structured framework for proactive, weather‐informed decision‐making in microalgal production. The findings show that forecast‐informed strategies substantially improve operational outcomes. Among the evaluated approaches, the GEOS‐5 model achieved the highest selection accuracy, correctly identifying the optimal strain and pond depth in approximately 84% of months, while the NLDAS‐2 climatology‐informed strategy achieved around 78% accuracy. Compared to a fixed cultivation strategy, both forecast‐informed approaches increased average monthly biomass yields by roughly 15%, with gains exceeding 40% in some months. These results underscore the potential of forecast‐guided decision‐making to enhance productivity and operational resilience, even under variable environmental conditions.

While this proof‐of‐concept study focused on one warm‐season strain (*P. celeri*) and one cold‐season strain (*T. striata*), the forecasting system is readily adaptable to a wider range of high‐performing strains, including warm‐adapted *Chlorella sorokiniana* USDOE 1412 and cold‐adapted *Monoraphidium minutum* 26B‐AM. Incorporating this broader physiological diversity will enable more nuanced decision‐making that accounts for strain–depth interactions, seasonal transitions, and environmental variability, supporting cultivation strategies tailored to specific production goals. All results presented here are based on simulations under idealized conditions, assuming optimal mixing, nutrient sufficiency, and the absence of contamination or biological disruptions. Field‐scale implementation may present additional challenges, such as variable light regimes, nutrient limitations, or unanticipated biological events. Therefore, future research will focus on three key directions: (1) expanding the model framework to include additional parameterized strains and conducting field trials involving multiple strains to evaluate system robustness under realistic conditions, (2) testing long‐term seasonal forecasts to support extended cultivation planning and strategy development, and (3) assessing system performance across geographically diverse sites to evaluate generalizability and regional adaptability. Overall, this study provides a promising approach for integrating forecasting into algal cultivation, demonstrating that monthly predictions can meaningfully enhance productivity, operational efficiency, and resilience. By offering a flexible and adaptive decision‐support tool, this study lays a foundation for further development of sustainable microalgae production strategies and the broader application of forecast‐informed cultivation planning in diverse environmental and operational contexts.

## Author Contributions

**Hongxiang Yan:** conceptualization, formal analysis, investigation, methodology, visualization, wiring – original draft. **Song Gao:** investigation, methodology, writing – review and editing. **Mark S. Wigmosta:** supervision, funding acquisition, project administration, writing – review and editing. **Andre M. Coleman:** project administration, writing – review and editing. **Ning Sun:** methodology, writing – review and editing. **Michael H. Huesemann:** supervision, writing – review and editing.

## Data Availability

NMME data can be accessed at https://www.ncei.noaa.gov/products/weather-climate-models/north-american-multi-model. NLDAS‐2 hourly data are available at https://disc.gsfc.nasa.gov/datasets?keywords=NLDAS. In‐situ meteorological data, excluding solar radiation, from the Mesa Gateway Airport station can be accessed at https://mesonet.agron.iastate.edu/request/download.phtml?network=AZ_ASOS. In‐situ solar radiation data from the AZMet Phoenix Encanto station are available at https://cales.arizona.edu/AZMET/az-data.htm. BGM simulation results supporting the findings of this study are available from the corresponding author upon reasonable request.
